# Complete Occlusion of the Vagina and Retention of Menses; Relieved by an Operation through the Rectum

**Published:** 1856-09

**Authors:** Frank Hastings Hamilton

**Affiliations:** Buffalo


ART. II.— *Complete occlusion of the Vagina and retention of Menses; relieved by an operation through the Rectum.* By FRANK HASTINGS HAMILTON, M. D., Buffalo.

Mrs. S., of ——, æt. 35, was brought to me Oct. 19, 1853. Six months before this she had miscarried when in her fourth month of pregnancy. The placenta was retained several days, inducing, by its decomposition, a violent metritis and vaginitis, which resulted, after a few weeks, in a complete closure of the vagina. Since then she has had occasional diarrhœas, and sometimes discharges of blood, especially about the time of her menstrual periods. She has also at such periods a sense of fullness in her loins, and a feeling of weight and pressure in her rectum.

I found the vagina completely closed to within about two inches of the vulva; the rectum and bladder seeming to be in immediate contact. There was no perceptible fullness of the abdomen, nor could I feel any fluctuation through the vagina.

Believing that I could not reach the uterus through the vagina without the risk of opening into either the rectum or bladder, and observing that her health was not greatly impaired, and especially, trusting that the period was near when she would cease to menstruate, I declined any surgical interference.

From this time for a period of about two years, she remained very much as when I first saw her, when she was visited by a traveling surgeon, who offered to give her immediate relief by an operation. The operation was made in the presence of her family physician, and consisted in an incision from the vagina directly through into the rectum, as the sequel showed, though at the time this fact was not known. The surgeon supposed he had entered the womb, but that it was empty. A hæmorrhage followed which was very near proving fatal. The wound into the rectum closed readily and she was left in the same situation as before except that she was much weakened by the bleeding.

March 6th, 1856, I was summoned by telegraph to see her, and I found her on the 7th suffering very greatly from the pressure of the accumulated menstrual fluid. For several months, at each period she had experienced great pain, but during the last four weeks there had been no relief except as it was afforded by chloroform in pretty full doses. She had been almost constantly confined to her bed; the enlarged uterus could be distinctly felt in the front of the belly and in the rectum. Examining with a curved probe introduced into the urethra and with the forefinger of my right hand in the vagina, I easily ascertained that the bladder was so deflected downward at the end of the vaginal *cul de sac*, that it would be impossible to reach the womb in this direction without opening the bladder. I therefore determined, as the only alternative, to open the womb from the rectum.

Placing my patient upon the side of the bed, I carried a trocar and canula upon the forefinger of my right hand into the rectum, and then thrust forward the trocar into the womb. On withdrawing the trocar there began to escape through the canula a dark brown, uncoagulated, inodorous fluid. I immediately pushed my finger along beside the canula and, without effort, tore open the womb in every direction, the walls of which were exceedingly thin. About one pint of this menstrual fluid was evacuated. With my finger I was able to explore the interior of the womb very freely, and I could not feel anything like a neck, the closure in that direction was so complete. On withdrawing my finger I noticed that the size of the opening which I had made was very much diminished by a contraction of the walls of the uterus.

Four months after this operation was performed, I learned that Mrs. S. had enjoyed uninterrupted health from the date of the operation, and an entire release from pain. She has menstruated regularly at each period, and without inconvenience.

